# Bioinformatics Study of *Aux/IAA* Family Genes and Their Expression in Response to Different Hormones Treatments during Japanese Apricot Fruit Development and Ripening

**DOI:** 10.3390/plants11151898

**Published:** 2022-07-22

**Authors:** Shahid Iqbal, Faisal Hayat, Naveed Mushtaq, Muhammad Khalil-ur-Rehman, Ummara Khan, Talat Bilal Yasoob, Muhammad Nawaz Khan, Zhaojun Ni, Shi Ting, Zhihong Gao

**Affiliations:** 1 College of Horticulture, Nanjing Agricultural University, Nanjing 210095, China; naveedmushtaq001@gmail.com (N.M.); nizhaojun@njau.edu.cn (Z.N.); shiting@njau.edu.cn (S.T.); 2 College of Horticulture, Zhongkai University of Agriculture and Engineering, Guangzhou 510225, China; maken_faisal@yahoo.com; 3 Department of Horticultural Sciences, The Islamia University of Bahawalpur, Bahawalpur 63100, Pakistan; muhammad.khalil@iub.edu.pk; 4 College of Food Science and Technology, Nanjing Agricultural University, Nanjing 210095, China; khanummaraft@gmail.com; 5 Department of Animal Sciences, Ghazi University, Dera Ghazi Khan 32200, Pakistan; tyasoob@gudgk.edu.pk; 6 Ayub Agricultural Research Institute, Faisalabad 38850, Pakistan; aliyan.nawaz22@gmail.com

**Keywords:** *Aux/IAA*, fruit maturity, hormones, Japanese apricot

## Abstract

Auxin/indole-3-acetic acid (*Aux/IAA*) is a transcriptional repressor in the auxin signaling pathway that plays a role in several plant growth and development as well as fruit and embryo development. However, it is unclear what role they play in Japanese apricot (*Prunus mume*) fruit development and maturity. To investigate the role of *Aux/IAA* genes in fruit texture, development, and maturity, we comprehensively identified and expressed 19 *PmIAA* genes, and demonstrated their conserved domains and homology across species. The majority of *PmIAA* genes are highly responsive and expressed in different hormone treatments. *PmIAA2, PmIAA5, PmIAA7, PmIAA10, PmIAA13, PmIAA18*, and *PmIAA19* showed a substantial increase in expression, suggesting that these genes are involved in fruit growth and maturity. During fruit maturation, alteration in the expression of *PmIAA* genes in response to 1-Methylcyclopropene (1-MCP) treatment revealed an interaction between auxin and ethylene. The current study investigated the response of *Aux/IAA* development regulators to auxin during fruit ripening, with the goal of better understanding their potential application in functional genomics.

## 1. Introduction

Auxin has a role in several cellular and developmental processes [1,2]. Auxin is a plant hormone that regulates apical control, phototropism, organ growth, and fruit maturation, among other biological processes [3,4,5]. Auxin stimulates diverse growth responses primarily through regulating gene expression [6,7,8]. Auxin/indole-3-acetic acid (*Aux/IAA*) [9], *Gretchen Hagen* 3 (*GH3*) [10], and small auxin up RNA (*SAUR*) are the three primary groups of early auxin response genes [11]. *Aux/IAA* genes belong to a vast gene family that may be found in a variety of plants, including 18 in papaya [12], 26 in grapes [13], 22 in pecans [14], and 27 in cucumbers [15]. Auxin is thought to influence plant development primarily through transcriptional regulation of the members of the auxin/indole-3-acetic acid (*Aux/IAA*), small auxin up RNA (*SAUR*), auxin response factor (*ARF*), and *Gretchen Hagen* 3 (*GH3*) gene families. [16,17,18,19]. Furthermore, these genes activate hormone signaling pathways that include salicylic acid (SA) [20], jasmonic acid (JA) [21], brassinosteroid (BR) [22], and ethylene [23].

*Aux/IAA* proteins retain four characteristic motifs, which are classified as domains I, II, III, and IV respectively. In this gene family, it has also been reported that proteins lack one or two of these domains [11]. Domain I includes a leucine repeat motif [LxLxLx], which inhibits transcription by communicating with a TOPLESS (TPL) repressor [24]. Domain II is the primary component that promotes the instability of *Aux/IAA* protein; therefore, it is thought to be the ubiquitin-proteasome protein’s degradation process [25]. Domains III and IV serve as binding sites for *Aux/IAA-ARF* hetero-dimers to assemble [26]. Previous research on *Aux/IAA* family genes have shown that they are crucial in controlling several aspects of plant growth [4,27,28]. Several *Aux/IAA* genes have been implicated in fruit growth and maturity, and are confirmed through earlier studies. The auxin response genes *Aux/IAA*1 and *Aux/IAA*2 were strongly up-regulated at the early stages of fruit development in strawberries (*Fragararia x ananassa*), but dramatically reduced throughout ripening [29]. The ethylene and auxin response gene *SlIAA3* was found to be expressed in several tissues of tomato (*Solanum esculenta*), however, it was shown to be more robustly expressed in orange, red, and mature fruits at the later stage of fruit maturity [30].

To better understand the *Aux/IAA* family in Rosaceae, we comprehensively investigated the entire *Aux/IAA* gene family of Japanese apricot (*Prunus mume*). The expression study revealed a distinct spatiotemporal expression pattern of the *Aux/IAA* genes, some of which had variable responses to maturation-related hormones, pointing to the functional characterization of auxin response genes involved in fruit maturation. The objective of this study was to see if and how *PmIAA* genes are differentially expressed during fruit ripening, and to determine if they may be used in future functional studies or as molecular breeding development targets. Our finding established the groundwork for further functional, molecular, and biochemical characterization of *Aux/IAA* activity in Japanese apricots, advancing our knowledge of auxin signal transduction during fruit development. Advances in auxin signal transduction will not only aid in the understanding of fruit tree genetics but will also give tools for designing and breeding novel features in Japanese apricot and other drupes.

## 2. Results

### 2.1. PmIAA Gene Characteristics and Phylogenetic Analysis

The *Prunus mume* genome (V 1.0) was used to identify the *PmIAA* genes in Japanese apricot. The gene sequences of *PmIAA* genes were retrieved from the *Prunus mume* genome. After removing repetitive sequences, 28 non-redundant putative genes were found and named *PmIAA*1–19. The details information on the genes including gene ID, length, exon number, chromosome, protein amino acid residues (aa), molecular weight (MW), pI, and subcellular localization are listed in Table 1.

**Figure 1:**
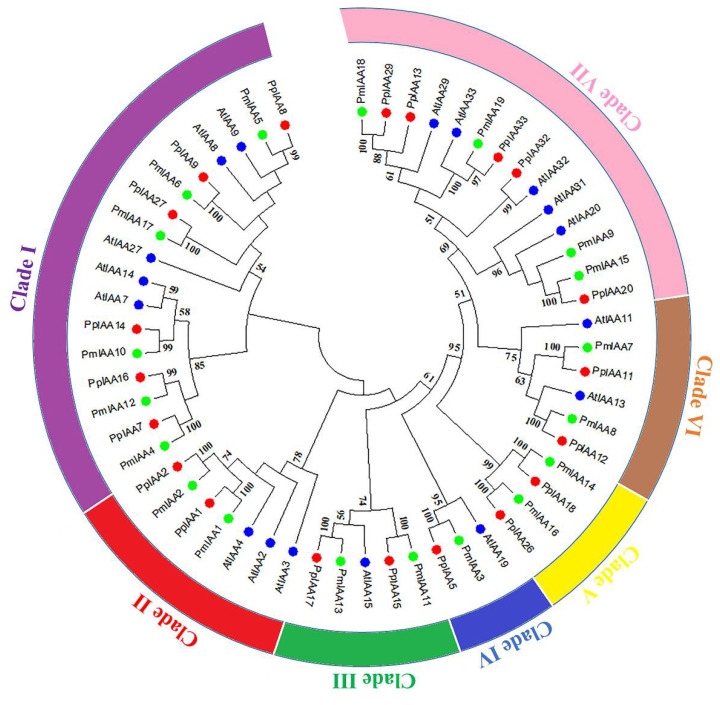
Phylogenetic analysis of PmIAA genes among different family members of Japanese apricot, peach, and *Arabidopsis* shown in green, red, and blue color dots, respectively. The number on the branches are the bootstrap values and are presented on each node. The outside circle of the phylogenetic tree shows different clades of the tree.

A phylogenetic tree based on *Arabidopsis* and *Prunus persica* was constructed using the neighbor-joining method (NJ) to investigate the evolutionary relationship of *PmIAA* proteins from *Prunus mume*. Based on *AtIAA* and *PpIAA* sequences, these genes were categorized into seven different clades (Figure 1). The maximum number of genes were grouped in clade I, while the minimum number of genes were grouped in clade IV. All the clades have interaction with peach and *Arabidopsis* except clade IV. The interaction between these clades showed their shared biological function among each other. The constructed phylogenetic tree is congruent with *Arabidopsis* and *Prunus persica*, suggesting diverse functions of the *PmIAA* genes.

### 2.2. Gene Structural Analysis and Motif Prediction

**Figure 2:**
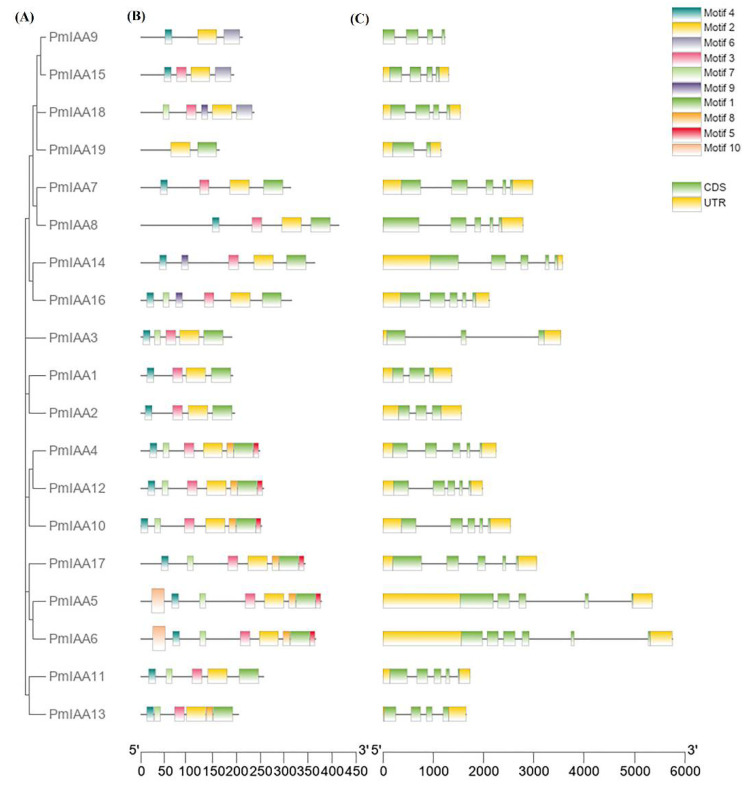
Analysis of phylogeny, gene structure and conserved motif of PmIAAs (**A**) The phylogenetic relationship of PmIAAs was constructed using MEGA 7.0 (**B**) conserved motif distribution of PmIAAs. Ten conserved motifs were labeled with different colors using MEME program (**C**) gene structural organization (CDS, UTR) of PmIAAs. The relative position is uniformly shown based on the Kilobase scale at the bottom of the figures.

A phylogenetic tree of *PmIAA* genes was constructed to further analyze their evolutionary relationships. To get deep insight and better understand the *PmIAA* gene structure, location of CDS, intron, and exon were identified using Gene Structure Display Server (GSDS; v2.0) (Figure 2). Therefore, based on coding sequence (CDS) and untranslated region (UTR) of IAA genes in *Prunus mume*, gene structure was determined and visualized in TBtools software. The finding revealed that the majority of the *PmIAA* gene members were mainly conserved, but have a lot of divergence from each other. Furthermore, most of the *PmIAA* genes showed fewer or more resemblances among the same clades.

To further investigate the diversity of IAA genes in *Prunus mume*, 10 conserved motifs were identified using the MEME online server. The majority of *PmIAA* genes have identical motif types and numbers, although there are variances in motif patterns among the same subfamily members. The results revealed that motifs 1, 2, 3, 4, and 7 exist in all *PmIAA* genes. The presence of identical gene architecture and conserved motifs in the same subfamily improves the phylogenetic tree’s accuracy. Furthermore, structural changes across subfamilies reveal the *PmIAA* gene family’s functional diversity.

### 2.3. PmIAA Genes Transcriptional Activity Is Regulated by Cis-Element

**Figure 3:**
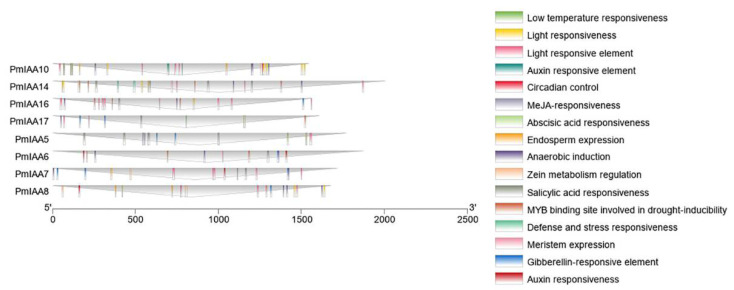
Various *cis*-elements were identified using the PlantCARE database. The different cis-elements biological terms are shown in different colors.

To further investigate the possible function of the *PmIAA* genes, >1500 kb sequences upstream from the translation initiation site of each *PmIAA* gene were selected and subjected to the PlantCARE database to identify putative *cis*-elements. The promoter region of the *PmIAA* genes contained 16 different types of *cis*-elements, including hormone-responsive elements, and development-response associated elements (Figure 3). The light-response and hormone-responsive elements were the most prevalent elements among all *PmIAA* genes. Furthermore, several additional stress response elements such as low temperature, anaerobic induction, and defense and stress responsiveness elements, and so on were also discovered, suggesting that they play an imperative role in coping with a variety of stresses. Auxin, Abscisic acid (ABA) and gibberellin-response elements were the most abundant hormone response elements, demonstrating their importance in hormone regulation.

### 2.4. Chromosomal Location and Synteny Analysis of PmIAA Genes

**Figure 4:**
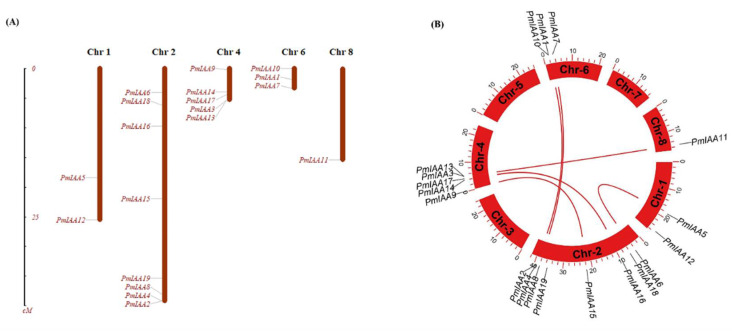
Chromosomal position and gene duplication analysis (**A**) The chromosomal position of each *PmIAA* gene was mapped according to the Japanese apricot genome (**B**) syntenic analysis for gene duplication showed pairs among different chromosomes The gene IDs on the chromosomes indicate the positions of centromeres and the scale on the circle is in Megabases.

The distribution of the chromosome was analyzed using MapInspect software. A total of 19 *PmIAA* genes were distributed randomly across the different chromosomal locations of the *Prunus mume* genome (Chr01–Chr08) (Figure 4A). Most of the chromosomes showed variation in terms of *PmIAA* genes. Chr02 showed the highest gene number (8 genes), while Chr08 had the lowest number of genes (1 gene). Chr01 represents two genes (*PmIAA5* and *PmIAA12*), Chr02 showed eight genes (*PmIAA2*, *PmIAA4*, *PmIAA6*, *PmIAA8*, *PmIAA15*, *PmIAA16*, *PmIAA18* and *PmIAA19*), Chr04 presented five genes (*PmIAA3*, *PmIAA9*, *PmIAA13*, *PmIAA14* and *PmIAA17*) and Chr06 showed three genes (*PmIAA1*, *PmIAA7* and *PmIAA10*), while Chr08 represent only one gene (*PmIAA11*). These genes were found on the upper and lower arms of the chromosome, suggesting that their uneven distribution might be attributable to chromosomal size and structural differences.

Subsequently, the tandem duplication of *PmIAA* genes was analyzed. As shown in Figure 4B, the *PmIAA* genes were differentially distributed in five out of eight J. apricot chromosomes. Among these genes, *PmIAA5–PmIAA6*, *PmIAA11–PmIAA13*, *PmIAA14–PmIAA16*, *PmIAA15–PmIAA9*, *PmIAA7–PmIAA8*, *PmIAA4–PmIAA10* and *PmIAA2–PmIAA1* exhibited gene duplication (Figure 4B).

### 2.5. Hormone Treatment Effect on the Expression of PmIAA Genes

**Figure 5:**
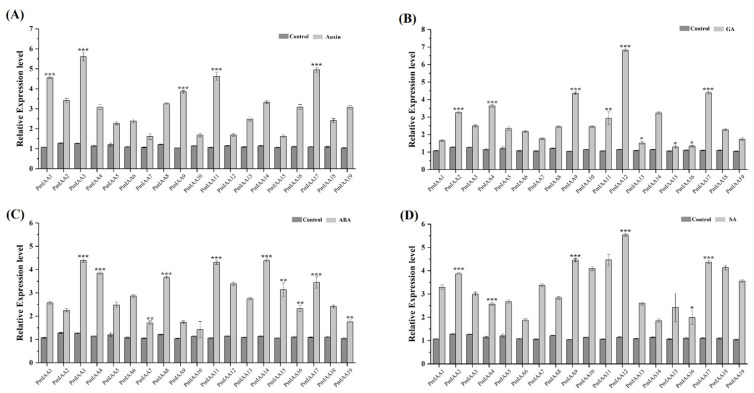
Expression of *PmIAA* genes in response to (**A**) auxin (**B**) GA (**C**) ABA (**D**) SA treatments was analyzed through qRT-PCR. The expression levels of *PmIAA* genes in control seedlings were set to a value of 1. The error bar represents the standard error of their biological repeats. Significant differences between control and treated samples are indicated in an asterisk (*). The sign * represents *p* ≤ 0.05, ** represents *p* ≤ 0.01 and *** represent *p* ≤ 0.001.

The primary hormones involved in fruit growth and ripening are auxin, ABA, GA, and SA. RT-qPCR was used to determine the expression pattern of *PmIAA* genes in response to these four hormones. Under the auxin treatment, the expression level of *PmIAA3*, *PmIAA11*, and *PmIAA17* was higher, while *PmIAA7 PmIAA10* and *PmIAA15* were lower (Figure 5A). Under GA treatment, the *PmIAA9*, *PmIAA12*, and *PmIAA17* showed higher expression, while *PmIAA13*, *PmIAA15*, and *PmIAA16* showed lower expression (Figure 5B). Under ABA treatment, the expression level of *PmIAA3*, *PmIAA11*, and *PmIAA14* were higher while *PmIAA7*, *PmIAA9*, and *PmIAA10* were lower (Figure 5C). While in the case of SA treatment, *PmIAA9*, *PmIAA11* and *PmIAA12* exhibited higher expression, while *PmIAA6*, *PmIAA14*, and *PmIAA16* exhibited lower expression (Figure 5D). The majority of *PmIAA* genes are phytohormones responsive and showed distinct expression patterns in response to various hormone treatments.

### 2.6. PmIAA Genes Expression after Exposure to 1-MCP

**Figure 6:**
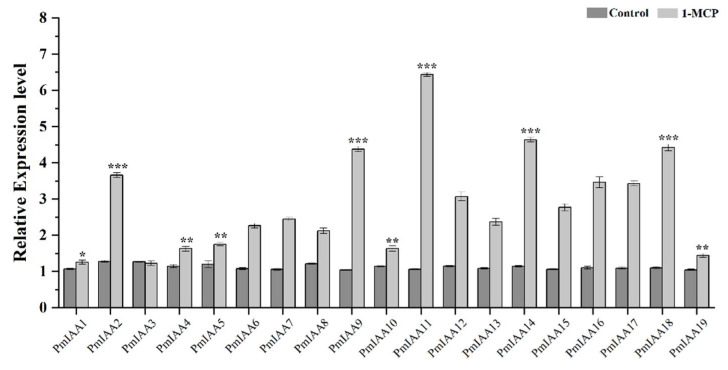
The expression of *PmIAA* genes in response to 1-MCP treatment was analyzed through qRT-PCR. The expression levels of *PmIAA* genes in control seedlings were set to a value of 1. The error bar represents the standard error of their biological repeats. Significant differences between control and treated samples are indicated in asterisk (*). The sign * represents *p* ≤ 0.05, ** represents *p* ≤ 0.01 and *** represents *p* ≤0.001.

Ethylene is the key hormone involved in fruit ripening. The expression of *PmIAA* genes involved in fruit ripening was examined using 1-MCP. The expression of *PmIAA2*, *PmIAA9*, *PmIAA11*, *PmIAA14*, and *PmIAA18* was significantly higher, while *PmIAA3* and *PmIAA19* showed lower expression (Figure 6). Overall, the expression of 1-MCP treated samples was higher than untreated samples.

### 2.7. Ethylene Production and Fruit Firmness

**Figure 7:**
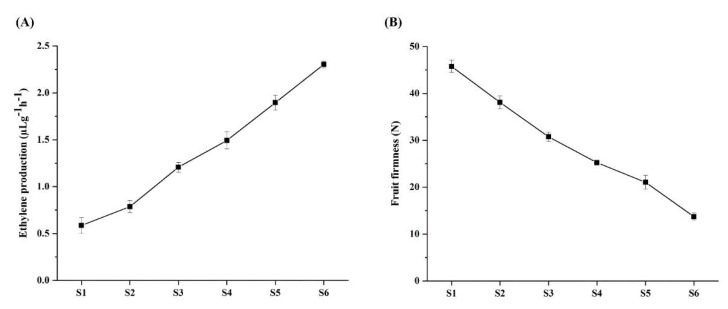
Changes in ethylene production (**A**) and fruit firmness (**B**). The horizontal axis represents the stages of fruit development. The error bar on data represents the standard error (±) of three individual repeats.

In the process of fruit ripening, ethylene production gradually increases from S4 to onward stages (Figure 7A). In the case of fruit firmness, the flesh firmness of the fruit gradually decreased during fruit development and ripening (Figure 7B). Overall, we can see that ethylene production becomes more with fruit ripening and the flesh of the fruit become softened gradually.

### 2.8. PmIAA Genes Expression throughout Different Stages of Fruit Development and Ripening

**Figure 8:**
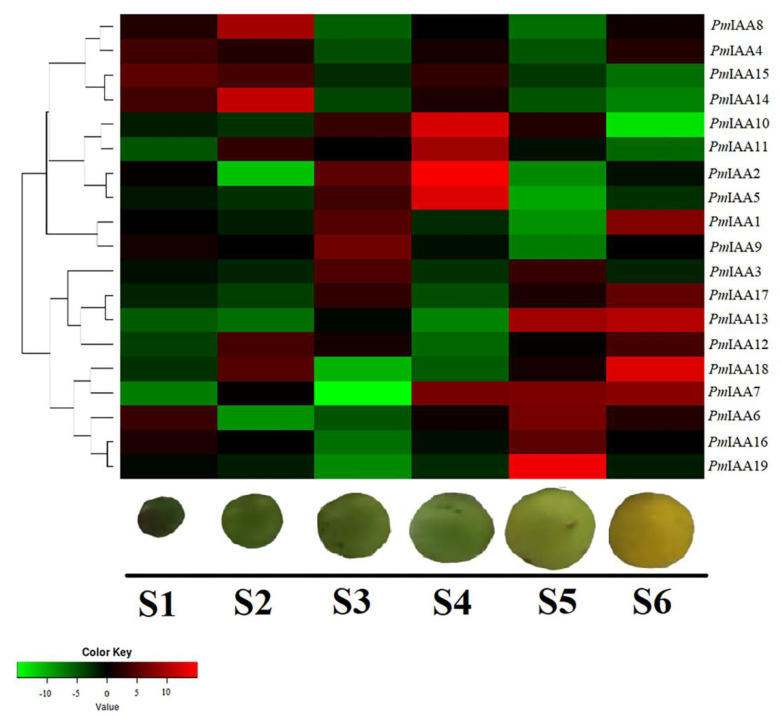
Expression profiles (hierarchical clustering) of *PmIAA* genes during different stages of fruit development and ripening. Color key shows the expression as red (up-regulated) and green (down-regulated) of the genes. Stages (S1–S6) and the fruit sample are shown under the Heatmap.

The expression level of all genes was analyzed during all stages from development to ripening. The expression of the genes is shown in cluster form (Figure 8). The expression of most genes was significantly changed from fruit development to ripening stages. The expression of *PmIAA2*, *PmIAA5*, *PmIAA10*, and *PmIAA11* was significantly higher at S4, while lower at S5 and S6 stages. At the S5 stage, the expression of *PmIAA19* was higher, while at the S6 stage, the expression of *PmIAA18* was higher. So, in light of these consequences, we can suggest that up-regulation of these *PmIAA* genes may contribute to fruit softening.

## 3. Discussion

Auxin is the main signaling molecule involved in different processes of plant growth and development [31,32,33]. Aux/IAA are the major proteins of auxin-mediated development that bind to ARF proteins to suppress or express the target genes [34,35]. Aux/IAA isolated genes mostly act to understand the plant metabolic functions and developmental process and have their role in early signaling [36,37,38]. In diverse plant species, the Aux/IAA gene families were identified and analyzed such as *Arabidopsis thaliana* [39], tomato [40], rice [10], and *B. rapa* [19]. However, in Japanese apricot, this information is quite little. To identify the mechanism of auxin involved in the development and ripening of Japanese apricot, a comprehensive collection of 19 *Aux/IAA* genes were identified and characterized, and their expression was analyzed.

For evolutionary analysis, a set of *Aux/IAA* genes with *A. thaliana* and peach species were taken and their phylogenetic tree was constructed and showed the distribution of genes in seven different clades/groups, suggesting that Aux/IAA in Japanese apricot were highly homologous to *Arabidopsis* and peach. The maximum number of the genes were presented in clades I and VII followed by clades II and VI. Relative phylogenetic research on Japanese apricot might give useful information regarding various biological functions and with earlier studies reported in *Arabidopsis* and rice [41,42]. In silico mapping showed that all 19 *PmIAA* genes were mapped on chromosomes 1, 2, 4, 6, and 8 of the genome. Syntenic analysis among *PmIAA* genes represents seven different segmental duplication genes, which have copies of 2 and 3 on different chromosomes. Further, motif analysis of *PmIAA* genes reveals changes in the conserved domain and motif structure, and most of the proteins were conserved in all domains, though someone was lacking. Their corresponding proteins from *Arabidopsis* also lack some domains, indicating that they are evolutionarily conservative in both species. While mutations or deletions of these domains can lengthen the life of these proteins when compared with other standards *Aux/IAA* proteins [43].

Fruit development is a complex process that involves the interaction of cell division, differentiation, and expansion in reproductive organs, and occurs in a spatially and temporarily organized manner [44]. In fruit plants, several reports suggested that auxin may be related to fruit development and maturation [45,46]. Auxin promotes cell division and elongation of un-pollinated resting ovaries, and is therefore considered to play a major role in fruit setting and development [47]. In plants, different hormones influence the degree of expression of IAA family genes [19]. According to our findings, several *PmIAA* gene transcript levels were influenced by various treatments. Promoter analysis revealed multiple recognized hormone response elements in the promoter region of most *PmIAA* genes, indicating that there is a crosstalk between several hormones in Japanese apricot, which is consistent with the change in *PmIAA* gene expression. In tomato, the member of the *Aux/IAA* gene family is involved in the formation of fruit. *SlIAA9* is continuously expressed in several tissues of the organ, and is rapidly stimulated by auxin [48]. In our study, auxin treatment dramatically increased the expression of *PmIAA9*, a homolog of *SlIAA9*. According to the previous study, ethylene production increased at the later stage of fruit development, and IAA concentration in mesocarp increased, indicating that auxin plays a regulatory role in controlling ethylene biosynthesis [49]. In our study, during the late ripening stage, the ethylene production was at its peak while the fruit firmness was lower at this peak stage. The considerable alterations in the expression of certain *PmIAA* family genes after treatment with 1-MCP suggested that auxin and ethylene had a strong interaction during fruit ripening. Our findings show how auxin affects fruit ripening and softening in a useful model.

The expression pattern of the *PmIAA* genes at different stages of fruit development revealed that the encoded proteins might have some similar specialized and redundant roles. Almost, all *PmIAA* genes were expressed, however at different stages of development, their level of expression varied substantially. *PmIAA2*, *PmIAA5*, *PmIAA7*, *PmIAA10*, *PmIAA13*, *PmIAA18*, and *PmIAA19* had considerably greater expression levels, suggesting that these genes are important for fruit growth and ripening. Many variables can contribute to differences in *Aux/IAA* gene expression, including tissue-specific auxin reception, cell type dependency, differential regulation of free auxin concentration, distinct patterns of auxin-dependent transcription, and post-transcriptional regulation [50]. Overall, the *Aux/IAA* gene expression data gathered in this work added to our understanding of auxin activity function during fruit ripening, and identified several prospective target genes for further investigation of putative regulatory mechanisms.

## 4. Materials and Method

### 4.1. Plant Material and Treatments

The Japanese apricot cv. Longyan plants are grown at the National Field Genbank of *Prunus mume*, Nanjing, Jiangsu, China were used as research material. At six various stages of growth and ripening, the maximum number of fruits were harvested, as S1 (first phase of fruit growth), S2 (light greenish color of the fruit), S3 (fruit ripening starts), S4 (no ethylene release during ripening), S5 (ethylene released at ripening) and S6 (fruit softening with more ethylene released). The fruits were then transferred to the laboratory and washed with deionized water to remove dust and microbes. For hormones (Auxin, ABA, SA, and GA), and 1-MCP treatment, fruit of the S3 stage was used. For hormone treatments, fruits were soaked in MS medium with various hormones such as Auxin, ABA, SA, and GA:10 uM each for 3 h. Further, the fruits were incubated for 16 h with 300 uL/L of the active component for 1-MCP treatment. For each treatment, three biological replications were performed with five fruits per replication. The fruits that were not treated served as a control. The samples were then frozen in liquid nitrogen and stored at −80 for subsequent testing.

### 4.2. In Silico Identification of PmIAA Genes in Japanese Apricot

The proteome sequences were retrieved from the *Prunus mume* genome [51] to identify IAA genes. The *AUX/IAA* conserved domain (PF02309) Hidden Markov model (HMM) was retrieved from Pfam [52], and used as a query to scan the *P. mume* proteome sequence. Multiple sequence alignment of full-length amino acid residues sequence was performed by Clustal W and removed the redundant sequences with the same gene. SMART [53] and Pfam [52] tool was used to predict the domains of *PmIAA* genes. Corresponding gene ids, their length, exon, and chromosome were analyzed from NCBI. Amino acid residues (aa), molecular weight (Mw), and pI were determined using the ExPASy tool [54], and the subcellular localization of the protein was performed using WoLF PSORT [55].

### 4.3. Phylogenetic Analysis Using Multiple Sequence Alignment

Clustal W was used to achieve multiple sequence alignment of *PmIAA* genes with default parameters. MEGA 7.0 was used to create a phylogenetic tree using Neighbour-joining algorithm methods with a 1000 bootstrap test as a replicate. The genes were classified into separate clades based on their ability to aggregate with *Arabidopsis (AtIAA)* and *Prunus persica (PpIAA)*.

### 4.4. Gene Structure and Motif Prediction

Gene Structure Display Server (GSDS 2.0) [56] was used to analyze the CDS, intron, and exon organization of the individual *PmIAA* gene by comparing the cDNA sequence with their genomic sequence. For motif prediction, MEME (Multiple Em for Motif Elicitation) [57] was used to analyze the conserved and sheared motif of the full-length protein sequence of *PmIAA* genes using the following parameters: minimum motif width- 6; maximum motif width- 50; and a number of motifs- 10. The combined result of GSDS and motif prediction was visualized using TBtools software (v 0.667) [58].

### 4.5. Cis-Element Analysis of PmIAA Genes

To analyze the putative *cis*-element and their activity in the promoter region of *PmIAA* genes, >1500 kb genomic sequence of transcriptional start site (ATG) were obtained from the *Prunus mume* genome. The sequences were consequently subjected to the PlantCARE database [59] to determine various *cis*-acting elements. The result of *cis*-elements was visualized using TBtools.

### 4.6. Chromosomal Location and Syntenic Analysis

The location of *PmIAA* genes on the chromosome was determined using the MapInspect software (v 1.0). The information on each chromosome length and position was obtained from the *Prunus mume* genome. The gene duplication of each IAA gene was analyzed, and the BLAST search was imported to MCScanX software. The synteny block within J. apricot was visualized in Circos, and the pair of the genes were considered segmental duplication gene pair.

### 4.7. Expression Analysis

RT-qPCR was used to evaluate *PmIAA* gene expression in response to hormones and 1-MCP treatment at six different stages of fruit development and ripening. Total RNA extraction and purification were performed by the method explained by Iqbal and others [60,61]. Primer Premier 5 was used to design the primers of the *PmIAA* genes and is listed in File S1. RP-II [62,63] was used as internal reference genes to standardize the reaction. RT-qPCR was accomplished by the method described previously [64,65]. The experiment was carried out with three biological replications (each replicate contains 5 fruits), and data were analyzed using the 2^−ΔΔCT^ method.

### 4.8. Ethylene Production and Flesh Firmness

To quantify the ethylene level of the fruit, 5 fruits per stage of each cultivar were taken and kept in a 1000 mL jar for 2 h. Gas chromatograph (Agilent Technologies, Santa Clara, CA, USA) was used to determine the ethylene content. The ethylene production rate was expressed as µL/gh. Fruit firmness (N) of each fruit was determined using a hand-held penetrometer (GY-4).

### 4.9. Statistical Analysis

Microsoft Excel (2016) was used for values calculation and Student’s *t*-test method was used for calculating differences among values. The significance levels are shown as; * represent *p* ≤ 0.05, ** represent *p* ≤ 0.01 and *** represent *p* ≤ 0.001. All experiments were repeated three times with three independent biological replicates.

## 5. Conclusions

The purpose of this study was to expand our understanding of *Aux/IAA* gene expression and hormone response during climacteric fruit maturation, and to identify several candidate target genes for further exploration of potential regulatory mechanisms. All the genes were conserved among *Arabidopsis* and peach; however, a little variation in the conservation and divergence of *PmIAA* genes was observed. Expression analysis revealed the involvement of *PmIAA* genes during fruit ripening and development affected by various hormone treatments. Our research opens up new avenues for research into the functional genomics of *Aux/IAA* genes, as well as new possibilities for plant genomics and breeding.

## Figures and Tables

**Table 1:** Physiological and biochemical properties of *PmIAA* genes.

Gene Name	ID	Length (bp)	Exon	Chromosomal Location	Amino Acid Residues (aa)	MW (kDa)	pI	Subcellular Localization
*PmIAA1*	LOC103334168	1140	3	LG6: 1870867..1872236	193	21.71	6.02	nucl: 9, mito: 3, chlo: 1, extr: 1
*PmIAA2*	LOC103323940	1312	3	LG2: 39181948..39183510	196	21.98	7.4	nucl: 9, plas: 2, chlo: 1, mito: 1, extr: 1
*PmIAA3*	LOC103327999	985	3	LG4: 5253934..5257475	190	21.2	7.29	nucl: 6, chlo: 5, cyto: 2, extr: 1
*PmIAA4*	LOC103323939	1243	5	LG2: 39166357..39168613	248	27.59	8.93	chlo: 11, nucl: 3
*PmIAA5*	LOC103335473	1771	7	LG1: 18408957..18414312	378	40.77	7.42	nucl: 11, cyto: 2, chlo: 1
*PmIAA6*	LOC103319419	1874	8	LG2: 4074649..4080394	366	39.72	7.53	nucl: 9, cyto: 2, chlo: 1, extr: 1, cysk: 1
*PmIAA7*	LOC103334408	1720	5	LG6: 3334525..3337512	314	32.77	7.34	nucl: 12, cyto: 1, cysk: 1
*PmIAA8*	LOC103323798	1681	5	LG2: 38082465..38085251	413	44.05	9.75	chlo: 8.5, chlo_mito: 7, mito: 4.5, nucl: 1
*PmIAA9*	LOC103327312	639	4	LG4: 174190..175421	212	23.3	5.92	nucl: 8.5, cyto_nucl: 5, chlo: 2, mito: 2, extr: 1
*PmIAA10*	LOC103334167	1548	5	LG6: 1861495..1864039	253	27.86	6.54	nucl: 11, chlo: 2, mito: 1
*PmIAA11*	LOC103340988	1139	5	LG8: 15328027..15329769	257	28.71	8.68	nucl: 13, cyto: 1
*PmIAA12*	LOC103318644	1219	5	LG1: 25493503..25495483	256	28.1	7.34	nucl: 6, chlo: 4, mito: 4
*PmIAA13*	LOC103328000	1001	4	LG4: 5264635..5266284	205	22.56	7.81	chlo: 6, nucl: 6, cyto: 1, extr: 1
*PmIAA14*	LOC103327830	2006	6	LG4: 3933212..3936787	363	39.9	8.58	nucl: 10, chlo: 2, cyto: 1, extr: 1
*PmIAA15*	LOC103322207	923	4	LG2: 21921723..21923036	196	21.25	7.37	chlo: 7, mito: 3, cyto: 2, nucl: 1, extr: 1
*PmIAA16*	LOC103320251	1563	5	LG2: 9688372..9690480	315	34.84	8.65	nucl: 9, cyto: 3, plas: 1.5, golg_plas: 1.5
*PmIAA17*	LOC103327885	1612	5	LG4: 4404671..4407734	343	37.36	7.8	nucl: 11, chlo: 1, cyto: 1, cysk: 1
*PmIAA18*	LOC103319708	1095	4	LG2: 6160523..6162071	237	26.8	9.77	cyto: 6.5, cyto_E.R.: 4, nucl: 3, extr: 2, chlo: 1, cysk: 1
*PmIAA19*	LOC103323477	897	2	LG2: 35394324..35395473	165	18.25	9.6	nucl: 6, cyto: 3, mito: 2, chlo: 1, plas: 1, vacu: 1

## Data Availability

Not applicable.

## Supplementary Materials

The following supporting information can be downloaded at: https://www.mdpi.com/article/10.3390/plants11151898/s1, File S1. List of primers used in this study.
